# Supplementary material for: Plasma Markers for Therapy Response Monitoring in Patients with Neuroendocrine Tumors Undergoing Peptide Receptor Radionuclide Therapy
Source: Cancers (Basel). 2023 Dec 6;15(24):5717. doi: 10.3390/cancers15245717 (PMC10741556; doi:10.3390/cancers15245717)
Supplement: Supplementary file 1 [file cancers-15-05717-s001.zip › Supplementary_Figure_S1.pdf]

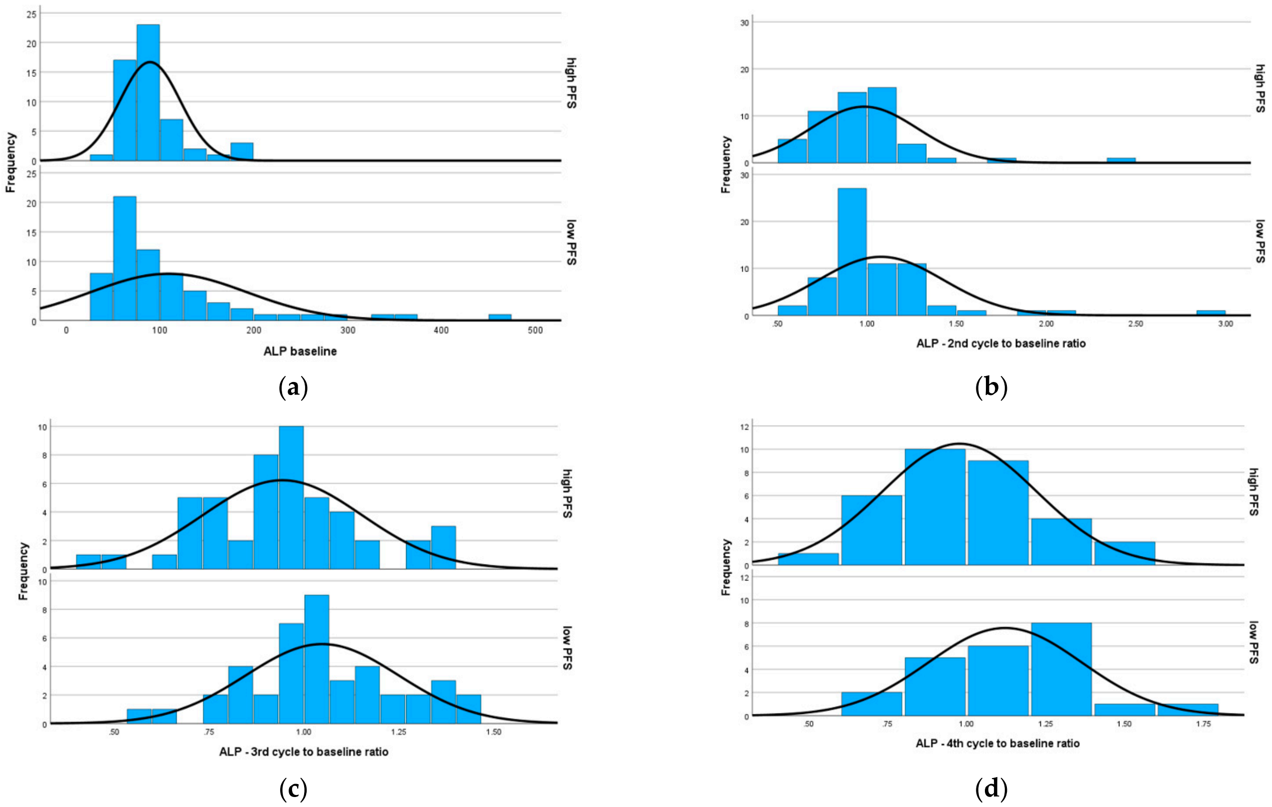

Figure S1. Histogram distribution of ALP variation between baseline (a), second cycle to baseline ratio (b) third cycle to baseline ratio (c) and fourth cycle to baseline ratio (d) separated in low vs high PFS groups.
