# Supplementary material for: Plasma Markers for Therapy Response Monitoring in Patients with Neuroendocrine Tumors Undergoing Peptide Receptor Radionuclide Therapy
Source: Cancers (Basel). 2023 Dec 6;15(24):5717. doi: 10.3390/cancers15245717 (PMC10741556; doi:10.3390/cancers15245717)
Supplement: Supplementary file 1 [file cancers-15-05717-s001.zip › Supplementary_Table_S1.pdf]

Table S1. Detailed patient characteristics of the 20 excluded patients.

Variable	n (%) or Median (Range)
Patient count	20
Age in years	66 (46 - 82)
Sex	
Men	9 (45%)
Women	11 (55%)
Primary location	
Small intestine	12 (60%)
Pancreas	2 (10%)
Colon/Rectum	2 (10%)
Lungs	2 (10%)
CUP	2 (10%)
Metastatic disease	19 (95%)
Metastatic spread	
Hepatic	19 (95%)
Lymphonodal	16 (80%)
Osseous	7 (35%)
Peritoneal	4 (20%)
Pulmonal	0 (0%)
Functional tumor	8 (40%)
Hedinger syndrome	2 (10%)
Grading	
G1	7 (35%)
G2	11 (55%)
G3	0 (0%)
Unknown	2 (10%)
Ki-67 index	5 (1 - 18)
Number of PRRT cycles	4 (2 - 4)
Previous treatment	
Operative resection	13 (65%)
Somatostatin analogues	16 (80%)
mTOR inhibitor	0 (0%)
Tyrosine kinase inhibitor	0 (0%)
Chemotherapy	2 (10%)
Local ablative therapy	1 (5%)
Radiation therapy	0 (0%)
Transcatheter arterial chemoembolization	2 (10%)
